# The future of basic science in orthopaedics and traumatology: Cassandra or Prometheus?

**DOI:** 10.1186/s40001-021-00521-x

**Published:** 2021-06-14

**Authors:** Henning Madry, Susanne Grässel, Ulrich Nöth, Borna Relja, Anke Bernstein, Denitsa Docheva, Max Daniel Kauther, Jan Christoph Katthagen, Rainer Bader, Martijn van Griensven, Dieter C. Wirtz, Michael J. Raschke, Markus Huber-Lang

**Affiliations:** 1 Institute of Experimental Orthopaedics and Osteoarthritis Research, Saarland University, Homburg, Germany; 2 Experimental Orthopedics, Department of Orthopedic Surgery, University of Regensburg, Regensburg, Germany; 3 Department of Orthopaedics and Trauma Surgery, Evangelisches Waldkrankenhaus Berlin Spandau, Berlin, Germany; 4 Experimental Radiology, University Clinic for Radiology and Nuclear Medicine, Otto-Von-Guericke-University Magdeburg, Magdeburg, Germany; 5 G.E.R.N. Research Center for Tissue Replacement, Regeneration & Neogenesis, Department of Orthopedics and Trauma Surgery, Medical Center - Albert-Ludwigs-University of Freiburg, Faculty of Medicine, Albert-Ludwigs-University of Freiburg, Breisgau, Germany; 6 Experimental Trauma Surgery, Department of Trauma Surgery, University Regensburg Medical Centre, Regensburg, Germany; 7 Department of Trauma-, Hand- and Reconstructive Surgery, University Hospital Essen, Essen, Germany; 8 Department of Trauma, Hand and Reconstructive Surgery, University Hospital Muenster, Muenster, Germany; 9 Department of Orthopaedics, Research Lab for Biomechanics and Implant Technology, Rostock University Medical Center, Rostock, Germany; 10 Department of Cell Biology-Inspired Tissue Engineering, MERLN-Institute for Technology-Inspired Regenerative Medicine, Maastricht University, Maastricht, The Netherlands; 11 Department of Orthopaedics and Trauma Surgery, University Hopsital Bonn, Bonn, Germany; 12 Institute for Clinical and Experimental Trauma-Immunology (ITI), University Hospital Ulm, Helmholzstr. 8/1, Ulm, Germany

**Keywords:** Basic science, Orthopaedics, Traumatology, Research agenda, Needs, Future

## Abstract

Orthopaedic and trauma research is a gateway to better health and mobility, reflecting the ever-increasing and complex burden of musculoskeletal diseases and injuries in Germany, Europe and worldwide. Basic science in orthopaedics and traumatology addresses the complete organism down to the molecule among an entire life of musculoskeletal mobility. Reflecting the complex and intertwined underlying mechanisms, cooperative research in this field has discovered important mechanisms on the molecular, cellular and organ levels, which subsequently led to innovative diagnostic and therapeutic strategies that reduced individual suffering as well as the burden on the society. However, research efforts are considerably threatened by economical pressures on clinicians and scientists, growing obstacles for urgently needed translational animal research, and insufficient funding. Although sophisticated science is feasible and realized in ever more individual research groups, a main goal of the multidisciplinary members of the Basic Science Section of the German Society for Orthopaedics and Trauma Surgery is to generate overarching structures and networks to answer to the growing clinical needs. The future of basic science in orthopaedics and traumatology can only be managed by an even more intensified exchange between basic scientists and clinicians while fuelling enthusiasm of talented junior scientists and clinicians. Prioritized future projects will master a broad range of opportunities from artificial intelligence, gene- and nano-technologies to large-scale, multi-centre clinical studies. Like Prometheus in the ancient Greek myth, transferring the elucidating knowledge from basic science to the real (clinical) world will reduce the individual suffering from orthopaedic diseases and trauma as well as their socio-economic impact.

## Introduction

Orthopaedic and trauma research is a gateway to better health and mobility, reflecting the ever-increasing and complex burden of musculoskeletal diseases and injuries. The field of basic science in orthopaedics and traumatology grows not only in Germany, but also across Europe and worldwide. Questions asked from orthopaedic and trauma surgeons to scientists who are involved in the many facets of musculoskeletal research and vice versa represent the exciting basis of fruitful interactions within this specific field.

Orthopaedic and trauma research is the key competence of the Basic Science Section (“*Sektion Grundlagenforschung*”, SGF) of the German Society for Orthopaedics and Trauma Surgery (“*Deutsche Gesellschaft für Orthopädie und Unfallchirurgie*”, DGOU). The SGF represents a multidisciplinary community of orthopaedic and trauma surgeons, biologists, biochemists, engineers, and veterinarians. Its members are devoted to orthopaedic and trauma research and aid in defining nationwide research policies in orthopaedics and trauma surgery through close cooperation of the corresponding committees and groups with the DGOU. Within the SGF, three networks exist, each of which has a specific focus: the network of musculoskeletal regeneration (MR-Net), the network of musculoskeletal biomechanics (MSB-Net), and the network for trauma research (NTF). The SGF plays also a key role in bringing together researchers, surgeons and other clinicians at the German Congress for Orthopaedics and Trauma Surgery (“*Deutscher Kongress für Orthopädie und Unfallchirurgie*”, DKOU). This annual meeting is considered the most important congress in the field of Orthopaedics and Traumatology in Germany and far beyond as it represents the largest of its sector in Europe with more than 10,000 attendees. At the annual congress, the SGF is responsible for organizing the basic research lectures and scientific poster sessions. Furthermore, the SGF is involved in the selection process of the recipient of the prestigious Basic Research Prize and honours outstanding scientific work with the annual Wilhelm Roux Award and several poster prizes, all of which are awarded during the common meeting.

**Fig. 1:**
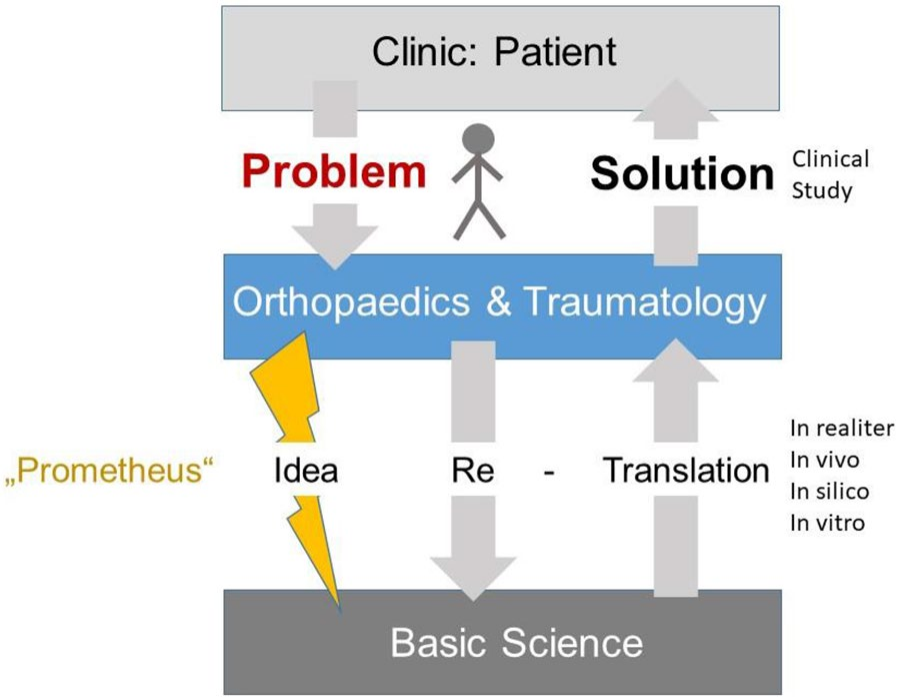
The Prometheus paradigm. The societies for orthopaedics and traumatology need to identify and define problems of the patient suffering from orthopaedic or (post)traumatic problems, which are so far diagnostically and therapeutically not satisfactorily resolved. Then, based on the “Prometheus” principle, a highly intelligent and innovative idea, evolved in the interdisciplinary discourse, may lead to a perfectly designed basic science study to reveal the underlying mechanisms. This can be realized, e.g. by a translational study with appropriate in vitro or in vivo (animal) modelling. The gained knowledge can then be translated back to the clinic and subsequently be evaluated for the final benefit of the patient

**Fig. 2:**
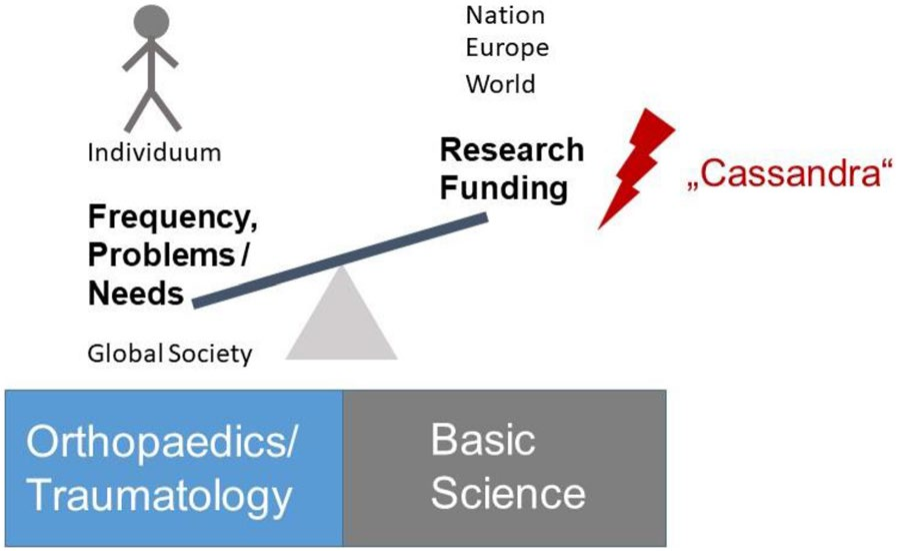
The Cassandra challenge. Disproportion of the high incidence as well as impact of orthopaedic diseases and trauma as opposed to the funding resources in the corresponding fields. In accordance to the “Cassandra” principle, the basic research societies in orthopaedics and traumatology might point to this imbalance without being heard by the surrounding environment and society—although they crusade for an improved quality of life and for improving the clinical outcome of the affected patients

Besides these national and global research efforts, more and more basic science questions arise which need to be addressed. However, far beyond the orthopaedic and trauma context, the lack of basic knowledge had been challenged already in ancient Greece by Prometheus who, endued with the highest degree of intelligence, brought the “fire” and “light” into the darkness of the human mind. An etymological analysis of the word “pro metheus” suggests that it derives from the Greek word Προμηθεύς, meaning “forethought” and “plan ahead”. Thus, Prometheus could function as a role model for the basic scientist in the field of orthopaedics and traumatology: by a wise study planning with a focussed standardized setting, the scientist is capable to enlighten enigmatic mechanisms, all of which may finally result in an adequate and satisfactory treatment of the patient (Fig. 1). In accordance, mechanism-driven trials, in which basic science-revealed specific mechanisms are targeted, are proposed to be more likely to show improvement in a heterogeneous trauma cohort [1]. On the other hand, basic science is also endangered to play the imposed role of Cassandra, the tragic Greek priestess who could accurately foresee the future but sadly was never believed. In free association, many basic research efforts and exciting findings will never make it to the bedside because they are neither perceived nor apprehended by the clinical-, funding- (Fig. 2), and political stakeholders and thus remain neglected. In this regard, during all stages of the career of both the scientist and clinician, a common language and understanding of the basic scientist and the clinician is often missing or underdeveloped. In case of the academic surgeon in the dual role as clinician and basic scientist, a framework has been proposed to accomplish the patient-centred trilogy of clinic, research, and teaching with a high degree of reliability and room for scientific and personal development [2]. In the case of the surgical/medical scientist such a dual role is rare but conceivable [3] and its further development is forward-looking (“pro-metheus”) and probably game changing. However, care has to be taken that such a medical scientist is not seen as a Cassandra. The balance between applied science and ethical medical treatment is delicate and needs to be guarded.

Orthopaedic and trauma research have overlapping interests, but some differences exist as well. Nevertheless, both fields are closely intertwined and therefore considered here. This paper addresses, from the different viewpoints of both orthopaedics and traumatology, the need of basic science, provides examples of cutting-edge research topics, elaborates on evolving methodologies to reach the state-of-the art, supports the need for high-quality animal research, and aims to define hot scientific topics and urgent needs that will have to be answered in the near and far future.

### Past research efforts in orthopaedics and traumatology: Cassandra or Prometheus?

The field of orthopaedics and traumatology addresses all musculoskeletal aspects of body support and motion. For example, a major task is the care for high-impact degenerative diseases such as osteoarthritis (OA) that challenge health-care systems globally. OA represents a high-burden non-communicable disease (NCD), its numbers significantly increase in terms of total burden and age-standardized disability-adjusted life-years (DALY), which considerably rose between 1990 and 2015 [4]. The hallmark of OA is the breakdown of the articular cartilage, although OA also affects all other tissues related to a joint. For many patients, pain and the slow and often immeasurable reduction in joint function are the main indicators of the disease and therefore also of the key to any potential conservative or surgical therapy. Orthopaedic research and treatment of OA has experienced significant advances over the past decades. Already in the 1960s it became clear that while pain may be relieved by intraarticularly injecting steroids, this treatment does not address cartilage loss and can even be harmful to the articular cartilage [5]. The discovery of growth factors, dating even back to the middle of the past century [6], was a crucial step as it led to the identification of factors that stimulate the articular chondrocytes to proliferate and to deposit extracellular matrix. This development can be regarded as a shining beacon enlightening orthopaedic and trauma research in the spirit of Prometheus, the mythological figure reflecting the quest for scientific truth and knowledge. The very first animal study on the effect of growth factors for cartilage repair conducted at the Charité hospital in Berlin was published in 1980 [7]. Nearly four decades later, the principle of applying growth factors for cartilage repair was finally tested in randomized-controlled clinical trials to potentially modify structural and clinical features of OA. The data revealed reductions in the loss of cartilage thickness over time when compared with placebo, however, without significant reduction in OA pain or other clinical parameters [8]. These extremely intriguing outcomes raise a number of burning questions to be addressed in the future; most importantly on the clinical relevance of such long searched-for structural changes and how to conduct future clinical trials of disease-modifying drugs that demonstrate effects not only on structure, but also on the clinical endpoints that matter to the patients [9]. This dilemma was already reflected by the classical remark of Henry Mankin, the renowned orthopaedic clinician scientist, who stated that the “cartilage does not yield its secrets easily, inducing cartilage to heal is not simple, … and progression to OA is sometimes so slow that we delude ourselves into thinking we are doing better than we are” [10]. Novel approaches for OA are being pursued to overcome this challenge, among which detailed investigation of topographical changes [11], OA pain [7, 8] and possibly contributing environmental factors [12], while also advancing gene-based [13] and other targeted approaches [14] as future therapies.

The field of trauma addresses any individuum since physical trauma can hit anybody and affect any region of the body at any time. Thus, trauma represents a major global burden of the past, presence and future [15]. Based on the highly interconnected organ systems (e.g. lung–liver, bone–kidneys, neuro-immune system) tissue injury to even a single bone or organ may affect via complex pathophysiological mechanisms various other tissues and thus in principle the whole body [16, 17]. Therefore, the field of trauma research comprises the whole human organism—from the first change on the sub-molecular level to the clinical bedside reality and far beyond into the society. As consequence, this cosmic broadness can barely be covered by the trauma research efforts of one nation, nor European- or even world-wide. Thus, trauma-related science has been attributed to be arbitrary, unfocused, fragmented and reduced quality research [18]. In clinical translation, numerous poorly designed studies have been performed, which addressed emergency, surgical and critical care management in various trauma settings with the main result: an urgent need for state-of-the art meaningful clinical studies remains and is further called for, especially to fill the gaps of corresponding guidelines [19–21]. However, clinical studies in trauma are rather complex and difficult to perform not at least due to the vast heterogeneity of trauma conditions, -patterns and -care [18]. Thereby, large numbers of patients need to be included to achieve statistically sound results. The involved pathomechanisms, which drive the healing and rehabilitation processes after trauma and in orthopaedics but also any complication directly associated with the quality of life, allegorize a “black box”, which is difficult to enlighten in a reasonable scientific, ethical and economic manner. Basic research in principle is capable to enlighten this “black box” of unknown mechanisms and provide the scientific rationale for an improved design of translational, clinically meaningful studies. These studies will return important insights in complex interactions and adjust the scientific models, which can subsequently be optimized.

However, it is also important not only to set the focus on external factors and trends affecting the evolution of trauma research but also to address—more alike blind Casandra’s approach—internal factors. Therefore, we should also question what could the field have done differently in the past and can we learn from other research disciplines? In this regard, uncertain career development paths for basic and clinical scientist in our field, ongoing separation of bench and bedside research, reduced industry–academic interactions, discouragement of innovative thinking, insufficient transdisciplinary networking, short-term research endeavours, as well as reserved public dissemination of meaningful results represent internal obstacles decelerating past and probably also present research efforts.

### Current hot topics in orthopaedics and traumatology

Since the areas of basic research within the field of orthopaedics and traumatology are numerous and steadily growing, only exemplary current hot topics can be delineated here.

In bone fracture healing, after initial inflammation, new cartilage and bone matrices are deposited that result in connecting the fracture ends. Currently, the recruitment of skeletal progenitor cells, vascular cells and subtypes of immune cells during the repair process as well as the direct, cell-to-cell and secretory cross-talk between exogenous and endogenous cells is extensively investigated [22]. Immunomodulation of musculoskeletal repair represents a very attractive area for novel therapeutic strategies: research efforts can lead to the identification of possible ways to spatio-temporal modulate specific immune cells to beneficially steer the repair process [23, 24]. Studies on the tight interplay of vascular, inflammatory and metabolic cascades during fracture healing are increasing [25]. Research in this area is critical to clarify the intimately intertwined cascades of tissue regeneration. Mesenchymal stromal cells (MSCs) represent also important immunomodulators, as they arise naturally at damaged sites but also when applied in a therapeutic approach. Regarding intercellular communication, current research addresses cellular exchange or even therapeutic application via extracellular vesicles [26, 27]. A current review on extracellular vesicles in musculoskeletal pathologies and regeneration serves as a timely example of a scientific network that is actively supported by the DGOOC [28]. Extracellular vesicles can also contain micro-RNAs that have been shown to be involved in many musculoskeletal diseases [29–31].

Regeneration of cartilage after trauma remains a hot topic since articular cartilage has a very poor capacity to repair itself and if healing occurs through fibrocartilage, it is characterized by insufficient structural and biomechanical properties. A better understanding of the cellular and molecular mechanisms of chondrocyte differentiation, phenotype preservation and the simultaneous response of chondrocytes to biochemical and biomechanical stress [32–34], may here provide insights into mechanisms that can guide chondrocytes and MSCs towards stable articular cartilage formation [35–37], which reflects the contemplations in the introduction about the impact of OA on the field of orthopaedics.

The awareness of the investigators on tendon, ligament, fascia and meniscal repair is also expanding towards understanding the intrinsic capacities of these tissues to heal [38] and the cell sources that participate in their repair [38–44]. Another fascinating exploratory field is on clarifying the impact of matrix composition, topography and biophysical properties onto the cells [45, 46]. Several recent studies have reported interesting novel data on the contribution of different cell types [47–53] as well as on the instructiveness of matrix properties on cell behaviour [46, 52, 54, 55].

Since it affects countless patients worldwide, intervertebral disc (IVD) regeneration is another basic science focus. Experimental studies explored the performance of different cell types when injected in IVDs [56–58]. Although there are significant advances in the basic understanding of IVD regeneration, this area is still in its prime and far from clinical translation when, for example, compared with the articular cartilage research. Since the IVD is a multilayered anatomical structure, namely nucleus pulposus, annulus fibrosus and the cartilage endplate, successful regeneration will require a simultaneous revival of all three tissues or functionally integrated in one implant [59]. Therefore, further basic research is needed on characterizing the molecular and cellular composition in homeostasis and degeneration of this unique structure [58, 60].

The orthopaedic and trauma fields have also recognized that repair proceeds at a different pace in young and healthy versus aged and degenerated/co-morbidity plagued musculoskeletal tissues and organs. Robust work has demonstrated that cellular niches and their endogenous progenitor cells have a profound impact during the age-related degenerative process [49, 61]. Hence, therapeutic principles have to be attuned to satisfactorily restore the structure–function of aged and injured tissues in the elderly. However, the search for the “fountain of youth” seems Prometheus-like, whereas Casandra may propose never to find such a source.

The mechano-biological pairing and the biophysics of cell–matrix interactions are essential in understanding the progression of musculoskeletal diseases and can also empower tissue regeneration. However, if these stimuli become abnormal they can prevent restoration and rather aggravate the disease. Current topics of interest within experimental orthopaedic biomechanics include mechanical testing of normal and diseased musculoskeletal tissues [62, 63], medical implant design and testing [64], tissue engineering [65–67] and translation of biomechanical into biochemical signals [68–71]. This research area will further optimize the biomechanical parameters of tissue-engineered implants and better understand cell- and drug-based therapeutic effects on mechanical behaviour at the tissue-level. In regard to tissue engineering, current biomaterial-related hot topics are “smarter” materials that are both degradable and able to control, steer or modulate biological responses and processes [72, 73]. The role of extracellular matrix in instructing biochemical cascades in cells has become rather evident [74, 75]. Also, materials that can closely mimic natural tissue properties and can navigate stem cell fates or exert immunomodulatory features are considered cutting edge [71]. The progress in developing such next generation biomaterials, that embrace the three-dimensional complexity of regenerating tissues as well as the interplay and optimal integration into the host tissue, can revolutionize biomaterial strategies in the near future.

With the rapid evolution of high-throughput, digital and information technologies, implementing system-oriented approaches to study musculoskeletal tissues, their diseases and repair processes become more and more realistic. In bone, the different cell subpopulations are well characterized. However, in cartilage, tendon, ligament, meniscus, and IVD, very interesting findings indicate an unexpected heterogeneity of cell subpopulations in these tissues (based on single cell RNA sequence data) [43, 48]. This may shift our understanding of the pathogenesis based on transition and prevalence of specific cell types during disease processes. Such research may result in defining cell subclasses that could be targeted to ameliorate disease progression versus cell types that can augment regeneration.

Another focus is set on platelets, especially after severe trauma. Platelets are fundamental to primary hemostasis, but become profoundly dysfunctional after polytrauma by unknown mechanisms, contributing to acute coagulopathy, severe bleeding and mortality. Circulating platelets are transformed into procoagulant balloons within minutes after trauma, and can release large numbers of activated microparticles/extracellular vesicles which coat leukocytes [76]. Furthermore, this study reports that the injury-induced danger molecule release (histone H4) functions as a driver of the procoagulant ballooning and subsequent innate immune response.

Concerning microvesicles (MVs), a recent study suggests that burn injury generates MVs, which allow skin keratinocytes to disperse bioactive substances. Applying diverse pharmacologic and genetic tools indicates that the optimal release of MVs is dependent upon the platelet-activating factor receptor [77]. Furthermore, MVs seem essential for transportation of metabolically labile bioactive lipids as cargo from cells in response to environmental stress. An important role of MVs concerning the complement C5a–C5aR1 axis was suggested in severely traumatized patients as well. C5a-induced MVs shedding from neutrophils decreased C5aR1 surface expression, while on the other hand profound inflammatory signals were induced, which may represent a key driver of the neutrophil dysfunction post trauma [78].

Trauma-induced emergency hematopoiesis characterizes the dramatic increase in the hematopoietic demand on the bone marrow to replace effector leukocytes upon their consumption during the inflammatory response to infection or injury. In experimental polytrauma, emergency hematopoiesis is mechanistically driven by the IL-1/MyD88/G-CSF-dependent pathway, resulting in the expansion of hematopoietic as well as myeloid-skewed and multipotent progenitor cells [79]. Furthermore, the role of specific inflammatory leukocyte subsets is currently ever broadened. In a trauma-induced sepsis model, endogenous intrinsic anti-inflammatory signals seem crucial to modulate the early monocyte/macrophages-driven inflammation by modifying their subset distributions [80]. In the clinical setting, the immunosuppressive properties of a neutrophil subtype (CD16^high^CD62L^low^) are gaining attention as causative and surrogate markers for increased susceptibility to infections post trauma [81].

Chronic inflammation in the elderly (“inflamm-aging”) has been proposed as major contributor to the decline in the regenerative capacity of the skeleton [82], mainly caused by skeletal stem/progenitor cell (SSPC) dysfunction [83]. A systemic and local proinflammatory environment was the major contributor of the decline in SSPC number and function resulting in cellular senescence [83]. Concerning muscle injury and regeneration, a metabolic cross-talk between macrophages and satellite cells has been defined, in which macrophage-derived glutamine preserves the function of satellite cells and thus provide a promising target [84].

Concerning remote organ injury after severe trauma, development of trauma-induced acute kidney injury (TRAKI) represents a role model for the impact of the immuno-pathophysiological trauma response [17]. In an ischaemia–reperfusion model of TRAKI single nucleus RNA sequencing of the kidneys allowed the characterization of various cell states during repair from acute injury: in the proximal tubule, a specific proinflammatory and profibrotic cell state was found that fails to repair [85]. “Full regeneration after amputation” could reflect a myth from Prometheus. However, in the ever exciting adult axolotl limb regeneration model, a novel regeneration-specific mitochondria-related cell cluster was discovered, and musculoskeletal cell populations supporting regeneration by providing energy were defined by modern tools (e.g. large-scale single-cell RNA sequencing and reconstructions of the dynamic single-cell transcriptome) [86].

Traumatic brain injury (TBI) is the strongest environmental risk factor for the accelerated development of neurodegenerative processes. Computational modelling provided insights into the cognitive decline and the presence of neurofibrillary tangles of the protein tau in the brains upon repetitive TBI [87]. The impacting high-strain rate deformation alone could induce tau mislocalization to dendritic spines and synaptic deficits in cultured hippocampal neurons which was inhibitable on the signalling level [87]. Thus, a mechanistic pathway directly relating mechanical deformation of neurons to tau-mediated synaptic impairments and a potential exploitable therapeutic approach to improve repetitive TBI consequences has now been provided [87]. A recently described molecular memory system (C–C-chemokine receptor type 5 (CCR5) signalling) was tested for its role in recovery after TBI [88]. Genetic and small molecule-based perturbation of CCR5 promotes functional recovery from TBI with preservation of dendritic spines and new patterns of cortical projections to contralateral pre-motor cortex [88]. Recently, the anti-inflammatory cytokine interleukin 13 (IL-13) was reported to accelerate functional recovery in murine TBI [89]. Furthermore, IL-13 reduced neuronal tissue loss, preserved white matter integrity, ameliorated the elevation of proinflammatory factors and reduced the number of proinflammatory microglia/macrophages after TBI [89]. Thus, IL-13 may present a potential immunotherapy to promote long-term recovery from TBI.

An interesting TBI model for basic science was developed and extensively characterized in Drosophila. Precise compression of the head using a piezoelectric actuator inflicts mild, moderate, or severe brain trauma in this Drosophila TBI model and provides a powerful tool to study the genetic system due to many conserved genes and pathways [90]. This tunable TBI model recapitulates mammalian injury phenotypes with severity-dependent ataxia, life span reduction, brain degeneration, cognitive decline and transient glial dysfunction. This model showed also stimulation of antioxidant, proteasome, and chaperone activity and thus underscores the ability of the stress response to mitigate TBI-induced brain degeneration [90].

### Evolving the methodology to reach the state-of-the art in orthopaedics and traumatology

The clinical care of orthopaedic and trauma patients in Germany can be classified as being at a state-of-the art level to which basic and applied medical and surgical research has significantly contributed. As mentioned above, a major disease burden in the orthopaedic field comes from OA, an age-related and/or trauma-induced multi-factorial, slowly progressing and primarily non-inflammatory degenerative disorder of the synovial joints culminating in the irreversible destruction of the articular cartilage [91]. The underlying molecular mechanisms have been addressed by basic research since many years. Breakdown of the collagen fibrillar network is a hallmark of cartilage extracellular matrix (ECM) degradation in OA [92, 93]. In this context, characterization of collagen triple helical domain structure, fragments thereof and folding mechanisms and kinetics was a basic research focus four to five decades ago. Commonly applied methods to do so were trypsin and pepsin digestion, SDS-PAGE, spectrophotometric measurements of triple helix formation, optical rotation and circular dichroism of collagen chains [94, 95]. Chromatography based on DEAE cellulose and amino acid composition analysis after HCl driven hydrolysis were common methods to characterize the molecular composition of collagen chains [96, 97]. This research focus on the molecular structure of collagens was followed by genetic based approaches, e.g. in the 1990s by the use of genetically modified mouse strains. Major methodologies to characterize the function and role of collagens and associated non-collagenous proteins in cartilage matrix integrity and turnover were skeletal staining, (immuno-)histochemical assays, in situ hybridization, TUNEL, SDS-PAGE and western blotting and ultrastructural analysis [98–100]. These methods were completed by northern blotting and RT-PCR-based gene expression analysis, biochemical methods such as application of recombinantly produced ECM proteins, solid phase binding assays, immunoprecipitation, immunoelectron- and immunofluorescence microscopy and 3D chondrocyte cell culture models. In addition, genetically modified mouse models for refined analysis of cartilage ECM proteins were designed [101–104].

Since the last two decades, high-end imaging methodology as atomic force microscopy (AFM), micro computed tomography (µCT) and nanoCT became more and more important for biomechanical and microstructural analysis of cartilage and (subchondral) bone. Indentation-type (IT) AFM is particularly useful to determine elastic properties of the chondrocyte pericellular matrix and cartilage ECM and the most sensitive method for detecting minute changes in cartilage biomechanics [105–108]. µCT application for topographical analysis of pathological structural changes of the osteochondral unit in translational animal models is state of the art [11]. To observe microstructural changes, i.e. in micro-channels in the subchondral bone zone and in the calcified cartilage zone, nanoCT analysis as high-end imaging modality is the preferred choice [107, 109]. In this line, the spatial organization of chondrocytes and its potential role in cartilage functioning and physiology became of special interest as reorganization and destruction of the basic spatial pattern during OA is important for responding to mechanical forces [110].

Increasing attention received the field of cartilage regeneration by employing chondrocytes, MCSs or recently chondrocyte progenitor cells (CPC), the latter residing in the articular cartilage. Regarding this focus, characterization of chondrogenic phenotype and chondrogenic differentiation capacity of MSCs and CPCs was of crucial importance. Sophisticated 3D cell culture techniques as cell pellet cultures, culture in alginate beads or fibrin/agarose gels were developed and refined together with the optimization of cell culture medium in order to maintain the chondrogenic phenotype and prevent dedifferentiation. One research group focused especially on chondrogenic differentiation of MSC and refinement of appropriate 3D culture systems [111–114]. Early key contributions on 3D culture of chondrocytes in order to prevent dedifferentiation and to keep the chondrogenic phenotype focused on the culture of chondrocytes in agarose [115, 116] and alginate beads [117, 118]. Identification of CPC (or MPC) in OA cartilage and their isolation, and establishing culture regimens was already described as early as 2004 [119] followed some years later by more extended work on the regenerative potential of CPCs [120, 121].

The genetic manipulation of MSCs became an attractive approach to produce therapeutic platforms for translational settings that aim at restoring articular cartilage defects. For that, gene transfer methods received increasing attention in order to improve the chondrogenic phenotype or proper differentiation of chondrocytes and progenitor cells. It was first reported in 2003 that AAV-based vectors can efficiently transduce and stably express foreign genes in articular chondrocytes, including chondrocytes of normal and osteoarthritic human articular cartilage, and MSC [122]. Numerous publications followed with ever-increasing refinement of gene delivery to the target cells/tissue [123–125]. Recent work focussed on delivery of therapeutic genes using specific biomaterial composites as scaffold-assisted gene therapy is considered as a highly promising tool to treat articular cartilage lesions upon direct delivery of chondrogenic candidate gene sequences [126, 127].

Concerning nationwide (trauma-)surgery-related research, a detailed survey was conducted in 1992 [128]. It revealed that the basic topics in the past mainly addressed transplantation immunology, implant biocompatibility, regulation of cell growth, control of of bone and cartilage growth, and sources of pain. Since then, a lot has changed and the importance of molecular biology for traumatology has steadily increased [129–132]. So far, main methods in surgical research addressed in principle three classical fields: in vitro, in vivo, and in real life, reflected by modern molecular biology-based methods [133, 134], animal experiments [135–138], and clinical studies, respectively [139]. The methodical spectrum in the field of molecular biology is immense, and novel, highly sophisticated methods are constantly added. Based on their widespread acceptance these serve today as a basic tool-box, including vectors for DNA cloning, restriction endonucleases and DNA hybridization, southern blotting, PCR, DNA sequencing and of course the analysis of highly polymorphic markers. For RNA analysis, Northern blotting, PCR, RT-PCR, qPCR, RT-qPCR, and, since a few years ago the digital droplet PCR (ddPCR) [140] are applied. Further methods are the transfection of eukaryotic cells, protein analysis, DNA cloning, gene mapping and identification, to name but a few. More complex cell cultures such as organ culture models and organoid models are utilized. New imaging methods like µCT, FIB-ESEM, TEM, CLEM, and live cell imaging also contributed to a better spatial resolution and understanding of posttraumatic degeneration and regeneration processes. With the help of molecular markers and the use of antibodies, e.g. also in GMP quality, it is now possible to mark cells and to trace their path within the body.

Moreover, with the help of numerical simulation tools, the behaviour of implants under different loading scenarios and microenvironments can also be predicted. The simulation includes the Finite Element Method (FEM) as well as multi-body simulations (MBS). While FEM is mainly used for field problems such as stress and strain calculations, implant micromovements as well as biological (re)modelling processes, MBS is used for dynamic problems such as gait analyses, investigation of muscle and joint forces or joint kinematics. Frequently in collaborative efforts with industrial partners, new implants have been developed and their design adapted to the respective clinical requirements [141]. Furthermore, surgical techniques have improved considerably in recent years [142]. Perioperative and rehabilitation quality management and evidence-based medicine have also found their way into surgical research [143–145].

New focal points in basic research have been established, e.g. in polytrauma research [146, 147], biomechanics on macroscopic [148, 149] and microscopic levels [150, 151], osteoimmunology [16, 152], and soft tissue- and wound healing [153]. Moreover, disturbance factors such as age [154–156] as well as co-morbidities [129, 157] such as diabetes, osteoporosis and obesity have been added to reflect a more realistic picture. Furthermore, promoting minimal invasiveness, recently, probe-based confocal laser-endomicroscopy combined with artificial intelligence (AI)-supported quantitative spatial data has been introduced in a proof-of-principle study as non-destructive optical biopsy to clinically detect early disease detection [158].

Together, these exemplary points indicate a paradigm shift in orthopaedic and trauma surgery research from pure clinical towards more basic research efforts to generate an improved understanding of the underlying mechanisms in trauma, inflammation, regeneration, degeneration, and repair processes. Based on a better micro- and molecular understanding, new diagnostic and therapeutic approaches will be developed and to be proven clinically.

### Why animal research in orthopaedics and traumatology?

In one traditional myth, Prometheus established the form of animal sacrifice practised in the ancient Greek religion. Such a sacrifice had been performed not only as service for a worshipped divinity, but also to accomplish some forseeing, i.e. to identify hidden truth and to predict the future from the appearance of inner organs. Thus, it is tempting to speculate that Prometheus was in some favour for animal research. In contrast, Cassandra could represent a person beset by doubt about the importance of animal research, but unheard by the surrounding people and society.

In translation to current orthopaedic and trauma research, both positions are advocated: pro- and anti-animal research although strict opponents to meaningful animal research remain a minority. However, it is undisputed that, like the German Research Foundation (DFG), orthopaedic and trauma research in Germany is committed to animal welfare and scientific validity and thus pursues the 3R principles [159]: replacement, reduction and refinement of animal experiments.

The necessity of animal modelling in trauma research is mainly deduced and justified by the complexity of the in vivo response to trauma and investigations on novel therapeutic interventions. In this regard, even the usage of organ-on-the chip technology and comptautional study designs cannot fully model the posttraumatic in vivo situation. Furthermore, the research of trauma-specific management, such as specific operation techniques or early resuscitation strategies and subsequent critical care are so far problematic (or even impossible) to model with the help of in vitro or in silico systems. Nevertheless, in the past, the validity of trauma modelling of clinical reality was questioned, e.g. in regard to genetic responses [160]. However, the murine models investigated indeed lacked a high simulation quality of the clinical setting. Therefore, multiple efforts have been undertaken to closer simulate the real world in trauma, burn and sepsis research [161] including international expert consensus initiatives to improve animal modelling [162] addressing among others the principle of “refinement”. Moreover, the use of a mouse- or pig intensive care unit seems to provide a higher degree of clinical simulation, validity and reliability [138, 163]. Furthermore, the better definition of the inflicted injury on well-defined anatomical regions helps to standardize the injury pattern and thus provides a better if not superior comparison with specific human situations [164]. Another development towards translational validity is the increasing consideration of various co-morbidities and their adequate modelling such as diabetes, osteoporosis, smoking, alcohol, atherosclerosis or chronic obstructive pulmonary disease in the context of trauma [165–167].

In the musculoskeletal system, delicate interactions between molecular, cellular, tissue and biomechanical levels exist that can only be incompletely modelled using for example computer-based systems (in silico) or in in vitro models. The need of animal research in orthopaedics may be exemplary highlighted by the cautionary tale of meniscal lesions and their close relationship to the articular cartilage and subchondral bone. The medial and lateral menisci -crescent shaped wedges of fibrocartilage located between the femoral condyles and the tibial plateau- perform important tasks to transmit loads and stabilize the knee joint [168]. Some hundred years ago, the menisci were regarded as functionless remnants of intraarticular leg muscles [169] and, consequently, they have been treated until the second decade of the last century with total meniscectomy. However, orthopaedic surgeons soon realized that such meniscectomized knees rapidly developed OA [170]. As this phenomenon can precisely be reproduced in both small and large animal models [171], it could have been foreseen if such studies would have been conducted before excising menisci in patients. Subsequently, techniques of meniscal refixation, repair, transplantation and replacement were evaluated and more and more refined over time with the ultimate goal of OA prevention [172, 173]. This narrative is in principle also related to the thalidomide disaster that caused numerous horrific birth defects in the human and that could have been prevented by more extensive preclinical testing in laboratory animals [174].

Today, the intricate relationship between meniscus, cartilage, bone, cruciate ligaments and others has developed into an entirely new field of research [175]. Although in vitro models immensely helped to elucidate molecular mechanisms and pathways involved in meniscal pathophysiology, the effect of a loss of meniscal tissue on the adjoining tissues and complex interactions within a biomechanically functional knee joint cannot be recapitulated. It is the testing of such clinically relevant interactions in (large) animal models that can help to elucidate these, often, intricate relationships. As a large animal knee joint is similar to that of humans in terms of joint anatomy, biomechanical function, cartilage and subchondral bone morphology [168], arthroscopic inspections and even reconstructive surgical interventions such as meniscal repair can be performed. Moreover, the postoperative course may be followed over relatively long periods, thus providing clinically relevant data that can only insufficiently be obtained by, for example, using a three-dimensional bioreactor culture with external forces applied.

In regard to “reduction” of animal experiments, orthopaedic and trauma research in Germany, Europe and worldwide has undertaken multiple efforts. An important step has been the formation of national networks such as the network for trauma research (NTF) or international research groups such as the Translational Large Animal Research Network (TREAT). These collaborative groups design, apply and perform common small and large animal studies and finally share tissues on a multi-organ level for synchronically answering different hypotheses. This results in an enormous reduction in animal numbers as if each hypothesis would have been investigated separately at each institution. For example, one recent pig study run by the TREAT group provided material for more then 10 collaborating research groups [176]. Similar efforts are undertaken at the trauma department at Aachen University [177, 178] or at the trauma collaborative research centre (CRC1149) at Ulm where multiple groups share organs from one mouse experiment. We consider these best practise examples of maximal reduction of animal numbers, performed at expert centres and shared by multiple clinical and basic researchers.

Concerning “replacement”, various ex vivo and in silico studies investigating interacting systems such as whole blood [179] or fracture healing [180, 181] are ongoing innovative developments even for first simulations of therapeutic principles. Furthermore, the publishing culture has changed not only in the field of orthopaedics and trauma research e.g. by following the ARRIVE guidelines and exact reporting of the experimental conditions [182]. Leading journals in the field endorse the use of the ARRIVE guidelines [183, 184]. The basic scientist in the field of orthopaedics and trauma is also encouraged to compare the results from the corresponding animal models with clinical reality to assess to which extend they match, e.g. immune and organ profiling in murine versus clinical polytrauma or to translate important scientific discoveries from the bench-to the operation theatre and back within the complex environment of the musculoskeletal system [185, 186]. Of course, in this research, the 3Rs principle must always be taken into account [159].

Taken together, basic research in orthopaedics and traumatology remains a valuable, important column of clinically meaningful research and is certainly committed to animal welfare and scientific validity.

### Future aims of basic science in orthopaedics and traumatology

In the near future, basic science in orthopaedics and traumatology will also be impacted by the revolution of technological improvement in materials and methods, especially due to computer-assisted techniques and a rapidly growing digitalization. On-site 3D printing technologies will be transferred to traumatology for research, education, and generation of individual (personalized) implants [187]. Due to the great variability of materials for 3D printing, further indication of this technology will be the treatment of bone and soft tissue defects with individually printed scaffolds [188]. Improvements in molecular imaging will increase the understanding of musculoskeletal pathologies. In the next ten years, artificial intelligence (AI) will be most likely introduced into clinical use in radiology and diagnostic imaging for the detection and classification of fractures and multiply injured patients [189]. Therefore, large databases for the use of AI have to be assembled and scientifically evaluated as AI will be a key feature in patient care in orthopaedics and traumatology. Trauma and implant registries will profit from digitalization with direct transfer of data leading to a more reliable quality. While established trauma registries lead to an improvement in outcome and quality assurance in major trauma in the last decade (e.g. CT in emergency room [190], prehospital intubation [191], prehospital tranexamic acid [192], future registries will focus on the ageing society (e.g. AltersTraumaRegister DGU) as the orthopaedic trauma surgeon will see less (young) multiply injured cases but an increasing number of geriatric patients. This will also need to be taken into account in in vitro studies [192]. The understanding and the treatment of sepsis and trauma associated immune-modulation will focus on the ageing patient as well [193]. As osteoporosis and malignant osteolysis will lead to an increasing number of pathologic fractures, finite element models for fracture prevention have to be introduced and evaluated as the CT-scan will be an increasingly used diagnostic tool for the geriatric patient in the future [194]. Hot topics of musculoskeletal regeneration will be tissue engineering of soft tissue i.e. muscle, tendon, cartilage and bone and their respective transitions [45, 195] with a focus on stem cells and extracellular vesicles [196] as well as further research on biodegradable implants and their clinical results. Biomechanical research will deal with the modulation of implant-derived debris, improvement of implant design and the application of gait analysis in the prevention of sports injuries.

In contrast to the revolution of new technologies and devices derived from basic science in orthopaedics and traumatology, the evolution in clinical life appears to be at a somewhat slower pace. Some authors claim that there is a stagnation in clinical translation of already known biomaterials, surface modifications and antimicrobial strategies for the control of biomaterial-implant-associated infections that has to be changed [197]. A challenge of clinical research in traumatology is the large variety of patients (e.g. fracture type, soft tissue damage, infection, age, bone quality, activity level, co-morbidities) with increasing treatment options offered by basic science (e.g. implant design, material, approach, molecular theragnostics) with a limited caseload, even in large centres. Therefore, international, industry-independent randomized-controlled trials have to prove the clinical relevance of new and existing devices and therapies on the market that derived from basic science.

### What structures do we need in the future?

Experts, progeny, money, and time—it could be as easy as this. But what are the real challenges and goals of orthopaedic and trauma research in the future?”*Tempora mutantur, nos et mutamur in illis*” which means that time is changing and changes us within: on the one hand the population is ageing, osteoporosis persists, fractures are becoming even more fragile; on the other hand, diagnostic means, treatment modalities and implant development are rapidly evolving. Digitalization as well as augmented and virtual reality will more and more become part of clinically relevant research and care. Nonetheless, the future of basic science in orthopaedics and traumatology primarily depends on the fascination of future young scientists and clinicians to join our exciting field. We will need to find the right way to inspire medical and natural science students early on for basic science in musculoskeletal research. Modern and contemporary working conditions will help to keep this research field competitive. We should not make the mistake to rely on past achievements but seek for concepts allowing for more protected research time.

A current barrier is sparse funding opportunities for orthopaedic and trauma research. Local intramural research programmes realized at several universities in Germany provide some benevolent start-up funding for young investigators. However, to date national and international research funding only offer limited opportunities to recruit larger peer-reviewed third-party funds due to a high demand but reduced supply. This gap will increase even more in the near future with EU funds for reseach in times of pandemic challenges considerably cut [198]. While the recent race for a vaccine during COVID-19 pandemic showed that EU countries are stronger if united than separated, the fact that (at the time of submitting this work) the United States added USD 10 billion since May 2020 to health crisis funding while the EU agreed in summer 2020 on slightly over USD 3 billion puts a spotlight on the importance and socio-economic impact of financial support for science [199]. The significance of understanding the pathomechanism of degeneration and injury and the need for high-end biomechanical research including modern robotic and simulation solutions must result in manifold funding options. Especially European funding for degenerative musculoskeletal and trauma research is largely missing. Excellent ideas of highly motivated and brilliant young investigators should be supported to boost future careers with lower funding threshold and without the need to prove extended preliminary work. The goal should be an easier entrance to the basic science world. In principle, such a path is proposed for “primary” applications at the German Research Foundation (DFG) but rarely established at other funding organizations.

These, together with fading industrial support, represent the “typical” means of funding that are known and used for decades. In other societal areas, other types of funding exist and are successful. Scientists in orthopaedics and trauma surgery should therefore seek to optimize their funding through these modern and creative funding possibilities. One can think of crowdfunding [200, 201] or setting up specific charity funds dedicated to orthopaedic and trauma research. Of course ethical and privacy issues needs to be taken into consideration [202, 203]. To enhance the effects of such funding strategies, the scientists should also enhance their exposure and outreach with a special emphasis on societal impact.

Although there will always be outstanding individuals—together we are stronger. Therefore, funding tools should also focus on support of research networks working on overarching questions and out of the box solutions. There must be a stimulus for cooperative research groups (18) such as the aforementioned initiatives like NTF, TREAT, MR-Net, MSB-Net, etc., rather than sole competition of few groups working in an isolated manner. Such cooperative efforts should be built up not just nationally but also European-wide or even globally.

Thereby, it should be strived for to develop an infrastructure for optimization of well-designed experimental animal studies by communicating planned projects prior to the start and invite external parties to participate. This may result in enhanced handling with the 3Rs and add to the translational value of studies as it may not only inspire groups already involved in basic science but also groups normally focussing on clinical work as well. Finally, also options should be explored to integrate projects with other (related and less-related) fields of research such as immunology, chronic inflammation, (cardio)-vascular research and identify in which trends (such as organoids and AI), the field lies behind.

The representatives of the umbrella organizations such as the SGF, DGOU, DGU, and DGOOC should further strengthen their work hand in hand to bring experts, progeny, money, and time for basic science in orthopaedics and traumatology and thereby in a long term to the patient care.

## Conclusion

Orthopaedic and trauma research in Germany, Europe- and world-wide gets a Cassandra-like, disproportional attention of funding in comparison to its relevance, to its importance for the individuum and socio-economic impact of related diseases and traumata (Fig. 2). Basic science in these fields addresses the whole conceivable spatio-temporal dimension of a human life with high-end structure–function tools. For the future, further development of networks and collaborative work, facilitated by overarching groups such as the SGF, help the multidisciplinary communities to define the urgent needs and research foci. Exciting new discoveries from the various fields of basic research will be translated from the laboratory to the clinical “real world”. Orthopaedic and trauma research should overcome any remaining boundaries between basic research and clinical reality, innovation and implementations in treatment, open research questions and available funding. The future of basic science in these fields can only be mastered by carefully listening to each other and intensified care for a common language between basic scientists and clinicians for a deeper understanding of the clinical mechanisms and therapeutic opportunities. Prioritized future projects will need to address a broad range of opportunities from AI, nano-technologies to large-scale, multi-centric clinical studies. Furthermore, Prometheus-like novel dissemination strategies to bring the light of basic science not only to the bedside but also into the awareness of society are mandatory. Only then, quality of life of the individual, suffering from orthopaedic diseases or trauma, and the global society will benefit from basic science efforts. Now is the time to act and to provide excellent and visionary programmes that will ensure the bright future of basic science in orthopaedics and traumatology.

## Data Availability

Not applicable since review.
